# Classification and Segmentation of Mucus Morphology Using Deep Learning During Diagnostic Nasal Endoscopy

**DOI:** 10.1002/alr.70004

**Published:** 2025-08-13

**Authors:** Dipesh Gyawali, Jonathan Bidwell, Sejal Shyam Bhatia, Akio Fujiwara, Thomas Mundy, Kayla Baker, Elena Bartolone, Dhara Patel, Edward D. McCoul

**Affiliations:** ^1^ Department of Otorhinolaryngology Ochsner Health New Orleans Louisiana USA; ^2^ Ochsner Clinical School University of Queensland New Orleans Louisiana USA; ^3^ Department of Otolaryngology Tulane University School of Medicine New Orleans Louisiana USA

**Keywords:** artificial intelligence, deep learning, diagnosis, mucus, nasal endoscopy, sinusitis

## Abstract

- A pretrained deep learning model can segment and classify mucus morphologies encountered during nasal endoscopy.
- Strong performance for different mucus morphologies supports the detection of sinonasal inflammation.
- This is the first study to objectively classify mucus morphology in nasal settings.

## 1 Introduction

The physical appearance of mucus during nasal endoscopy (NE) has potential diagnostic significance during the evaluation of rhinosinusitis [1]. Clinicians rely on visual clues to inform their diagnostic process, but considerable variability exists due to subjective judgment [2]. Even with specialized training, interpretation of findings can be inconsistent and subjective [3].

Prior work has described the development of a deep learning tool that can run in real time to identify important landmarks encountered during NE [4, 5, 6]. The present study aims to advance the segmentation of structure, particularly mucus and its morphologies, to reduce variability in the diagnostic process.

## 2 Methods

### 2.1 Data Collection and Preparation

A convolutional neural network (CNN) based on the YOLOv11 nano model [7] was trained on 3536 images (1024 × 768 pixels) collected from 2014 to 2025 during routine NE at a tertiary rhinology clinic by a single clinician (E.D.M.) using digital video endoscopes (Karl Storz, Tuttlingen, Germany). Images encompassed key anatomical landmarks, including the turbinates, middle meatus, and sphenoid recess. Frames were selected regardless of mucus quantity or prominence to reduce selection bias and reflect real‐world variability.

Manual segmentation and morphology classification were performed by trained physicians (K.B., E.B., D.P., E.D.M.) using open‐source COCO‐Annotator labeling software (https://github.com/jsbroks/coco‐annotator). Morphologies were chosen on the basis of clinical relevance and visual distinction.

### 2.2 Two‐Stage Model Architecture

**FIGURE 1:**
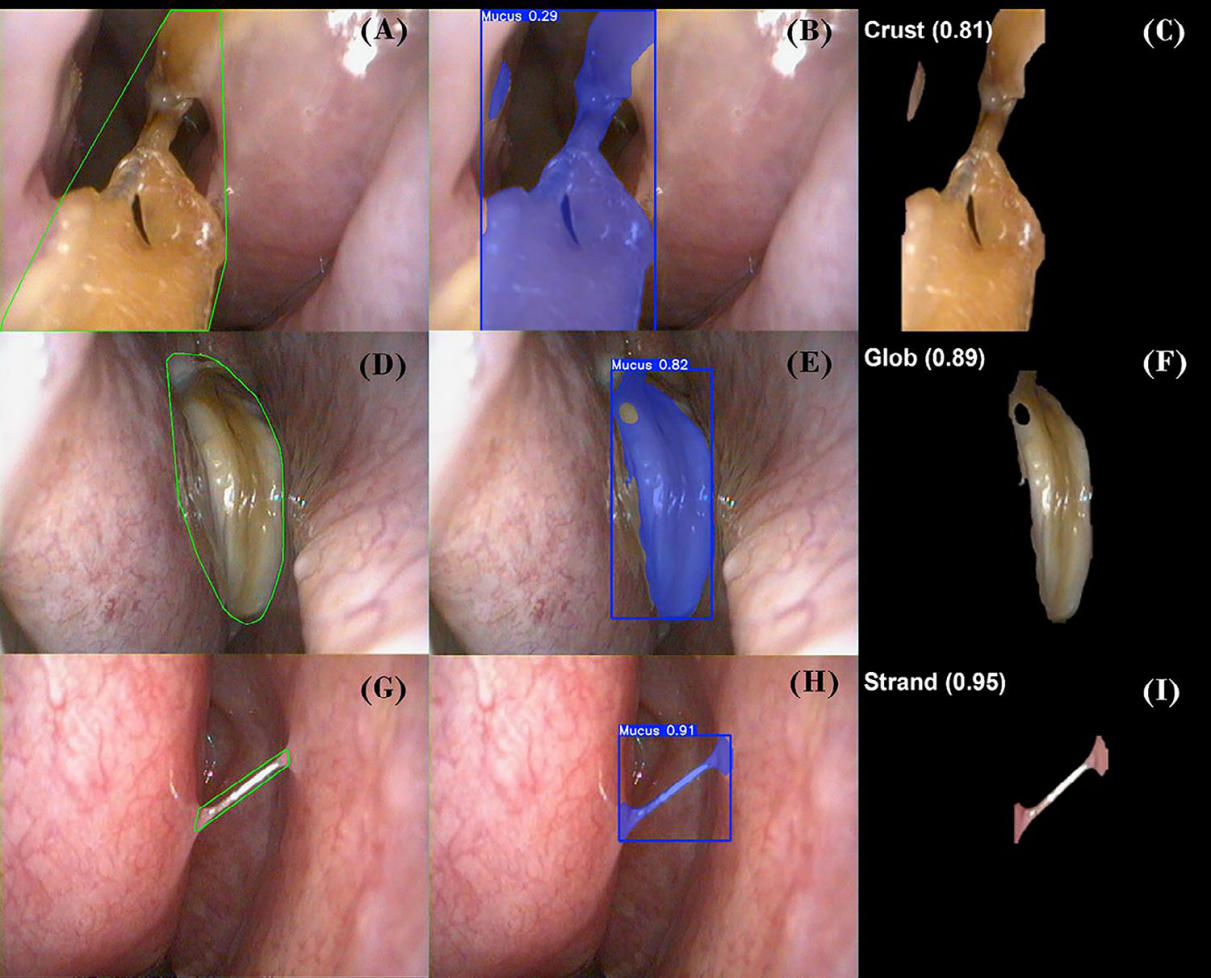
Examples of segmentation and classification outputs for crust, glob, and strand, grouped by detection type. (A) Ground truth: crust, (B) mucus detection, (C) classification: crust with probability score, (D) ground truth: glob, (E) mucus detection, (F) classification: glob with probability score, (G) ground truth: strand, (H) mucus detection, (I) classification: strand with probability score.

Initial tests of a single‐stage model that directly segmented and classified mucus morphologies yielded low accuracy (*F*1 < 0.60) due to background noise and irrelevant visual features. To improve performance, we implemented a two‐stage architecture (Figure 1). The model was trained to localize mucus regions using binary masks, excluding non‐diagnostic elements such as glare, blood, and surrounding anatomy. Then, using ROI masking [8], segmented mucus regions were cropped and passed to a separate classification model trained on crust, glob, and strand morphologies. Glob was an accumulation of mucus in an amorphous configuration lying on a mucosal surface. Crust was solid matter representing dried mucus. Strand was a fine entrail of mucus suspended across the lumen with the ends affixed to opposing mucosal surfaces.

This modular approach improved focus, reduced noise, and increased accuracy, enabling real‐time, morphology‐specific analysis.

### 2.3 Training Setup and Evaluation

The model was trained using a patient‐level split, with a separate held‐out test set to ensure no patient overlap and prevent data leakage. Within the training set, a 90/10 split was used for validation and hyperparameter tuning to maximize training efficiency while preserving generalization. Early stopping (patience = 100) was applied during training, which was conducted for up to 400 epochs with 5‐fold cross‐validation. Hyperparameters included learning rate (1 × 10^−2^), momentum (0.937), batch size (32), and optimizer (Adam). We performed augmentation with horizontal flip (0.5), mosaic (1.0), resize (640 × 640), and translation (0.1). Training and inference were performed in real time on an NVIDIA RTX 5500 GPU.

## 3 Results

Patient‐level stratification allocated 362 patients (3124 images) to training and 92 patients (412 images) to testing, ensuring no patients overlap. Mean age was 61 ± 1.2 years, with 42% males and 58% females. The training set contained 1213 crust, 2590 glob, and 1597 strand instances; the testing set contained 146 crust, 406 glob, and 217 strand instances. Images could contain multiple morphologies.

**FIGURE 2:**
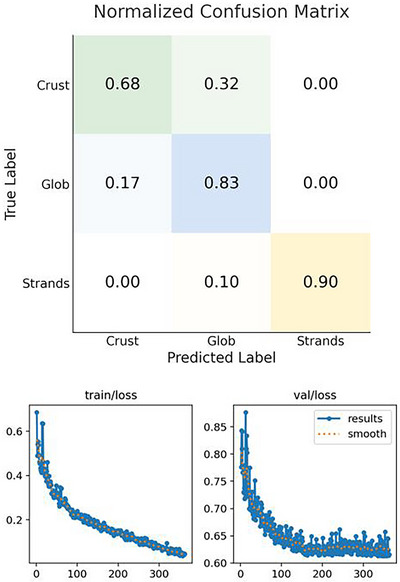
Normalized confusion matrix and training and validation loss curve (*x*‐axis shows number of epochs and *y*‐axis shows the loss value).

The model achieved robust accuracy for strands (*F*1 = 0.95, sensitivity = 0.90, precision = 1.00) and globs (*F*1 = 0.73, sensitivity = 0.83, precision = 0.66), with moderate performance for crusts (*F*1 = 0.73, sensitivity = 0.68, precision = 0.80) (Figure 2). Principal errors included 32% crust‐to‐glob misclassification and 17% glob‐to‐crust, whereas strand misclassification was minimal (10%). Specificity was highest for strands (1.00), followed by crusts (0.83) and globs (0.58). Mean inference time was 25.6 ms (39 fps).

## 4 Discussion

The two‐stage architecture—separate segmentation and classification networks—improved accuracy over a one‐stage baseline by removing irrelevant background and isolating mucus before classification. The classifier achieved strong performance for strands and globs and moderate performance for crusts, which often resemble globs under endoscopic lighting. Inference speed supports clinical use through live on‐screen overlays, whereas the model's compact size enables edge deployment.

These mucus morphologies were chosen because of distinct pathologies that they might represent. Globs are consistent with thick mucus from acute or chronic sinusitis. Crusts represent dried purulent mucus as encountered in advanced chronic rhinosinusitis. Strands represent non‐purulent mucus, as in nonbacterial rhinitis. Clinical correlates of these morphologies will be established in future study.

Recent studies of AI in NE have achieved an *F*1 of 0.85–0.95 for polyp segmentation and a Dice of ∼0.74 for nasopharyngeal carcinoma, with reported sensitivities up to 98% [9]. Although these tasks involve more visually distinct targets, our system achieved comparable performance (*F*1 = 0.95 for strand, 0.73 for glob/crust) despite the greater visual subtlety of mucus morphology. These results position our approach alongside leading segmentation tasks in other endoscopic applications.

The model was constrained by training on static frames with a limited dataset. However, inference can be applied to video streams on a frame‐by‐frame basis without modification, with comparable results when image quality is consistent. Crust classification remains a challenge due to overlapping features and lighting artifacts. In real‐world settings, variability in hardware, operator skill, and lighting conditions may affect performance. Future study will validate the system using video‐based input and additional data and evaluate its impact on diagnostic consistency and workflow integration. Additionally, subsequent work will apply this algorithm in real time to a clinical population as a clinical decision‐support tool.

This is the first study to apply a CNN‐based system for automated classification of mucus morphology during diagnostic NE. Automated mucus classification can standardize diagnostic interpretations and may improve clinical care. External validation is needed to quantify clinical outcomes and training impacts.

## Conflicts of Interest

Edward D. McCoul (EDM) is a consultant for 3D Matrix, Advanced Rx, Optinose, Sanofi/Regeneron, Stryker, and Zsquare. The other authors declare no conflicts of interest.
